# Impaired olfaction post-coronavirus disease 2019: a systematic review of smell recovery predictive factors

**DOI:** 10.1186/s43163-022-00271-5

**Published:** 2022-07-07

**Authors:** Nunki Puspita Utomo, Arin Dwi Iswarini

**Affiliations:** 1 Faculty of Medicine, Duta Wacana Christian University, Yogyakarta, Indonesia; 2 Otorhinolaryngology-Head and Neck Surgery Department, Bethesda Hospital, Yogyakarta, Indonesia

**Keywords:** COVID-19, Olfactory disorders, Predictive factors, Recovery of function

## Abstract

**Background:**

The devastating coronavirus disease (COVID-19) pandemic seemed not yet to cease. Numerous studies regarding its typical sign and symptoms have been done, presenting one of the most promising predictors of the infection: olfactory dysfunction. Although not life-threatening, the symptom could decrease one’s quality of life, especially if persistent throughout their entire life. Among the countless literature regarding loss of smell, only limited studies denote predictors of smell recovery. This systematic review aimed to bridge the knowledge gap of olfactory impairment prevalence and recovery predictors in people with COVID-19.

**Methods:**

This review was carried out through journal databases, including PubMed, Science Direct, Google Scholar, and medRxiv. Literature published from 2020 to 2022 that complied with the inclusion and exclusion criteria was retrieved, scanned for duplicates with Zotero, and reported using the *Preferred Reporting Items for Systematic Reviews and Meta-Analyses Protocols* 2020 (PRISMA) guidelines.

**Results:**

Of the 2243 studies, seven were assessed with the Prediction model Risk Of Bias ASsessment Tool (PROBAST) to analyze the risk of bias, which five cohort studies deemed relevant. Olfactory dysfunction, olfactory recovery, and its predictive factors are noted. This review is registered in PROSPERO (Registration Number CRD42022318412).

**Conclusions:**

No clinical markers predicted the recovery of olfactory dysfunction, but patients who are more likely to recover are associated with younger age, female sex, and having COVID-19-related symptoms such as nasal congestion and trigeminal sensation. Modifiable factors are still dubious in predicting the olfaction recovery.

## Background

As the coronavirus disease 2019 (COVID-19) emerges as a global pandemic, researchers worldwide have determined to study the symptomatology. One symptom allegedly becomes the most prevalent of all chemosensory symptoms, olfactory dysfunction [1]. Anosmia—disappearance of olfactory function—could present as the only symptom of COVID-19 [2, 3], in which the individuals presenting influenza-like symptoms are 6–10 times more likely to be COVID-19-positive [3, 4]. However, the chance of olfactory dysfunction to persist was up to 24% in more than 7 months post-COVID-19 onsets—with 23.3% of the patients complaining of complete anosmia 35% [5], which causes slightly morbid inconvenience in 1 out of 3 individuals [6]. The inconvenience was described in many degrees, including a lower drive to cook and eat resulting in weight loss, increasing social anxiety and anxiety due to cleanliness, and one that could be potentially life-threatening: failure to detect hazards such as gas and fire, thus provoking more anxiety when alone [7].

Despite the trend toward a more declining rate of COVID-19, the term “Long COVID” translated as post-COVID-19 persistent syndrome seemed to include smell and taste dysfunction [8], thus provoking more burden in the daily life of 1 out of 5 COVID-19 survivors [9], which results in a large number of people living with long-term morbidity [10] even though most of the individuals affected by it are diagnosed relatively late [11].

Much is known about the risk factors and symptomatology of COVID-19, but little has been discussed about the recovery of the COVID-19 sequelae, especially olfactory function.

## Methods

### Study design

A systematic review was conducted to overview the prevalence and prognosis studies. All studies are appraised with the Prediction model Risk Of Bias ASsessment Tool (PROBAST) [12].

### Searching strategies

**Fig. 1:**
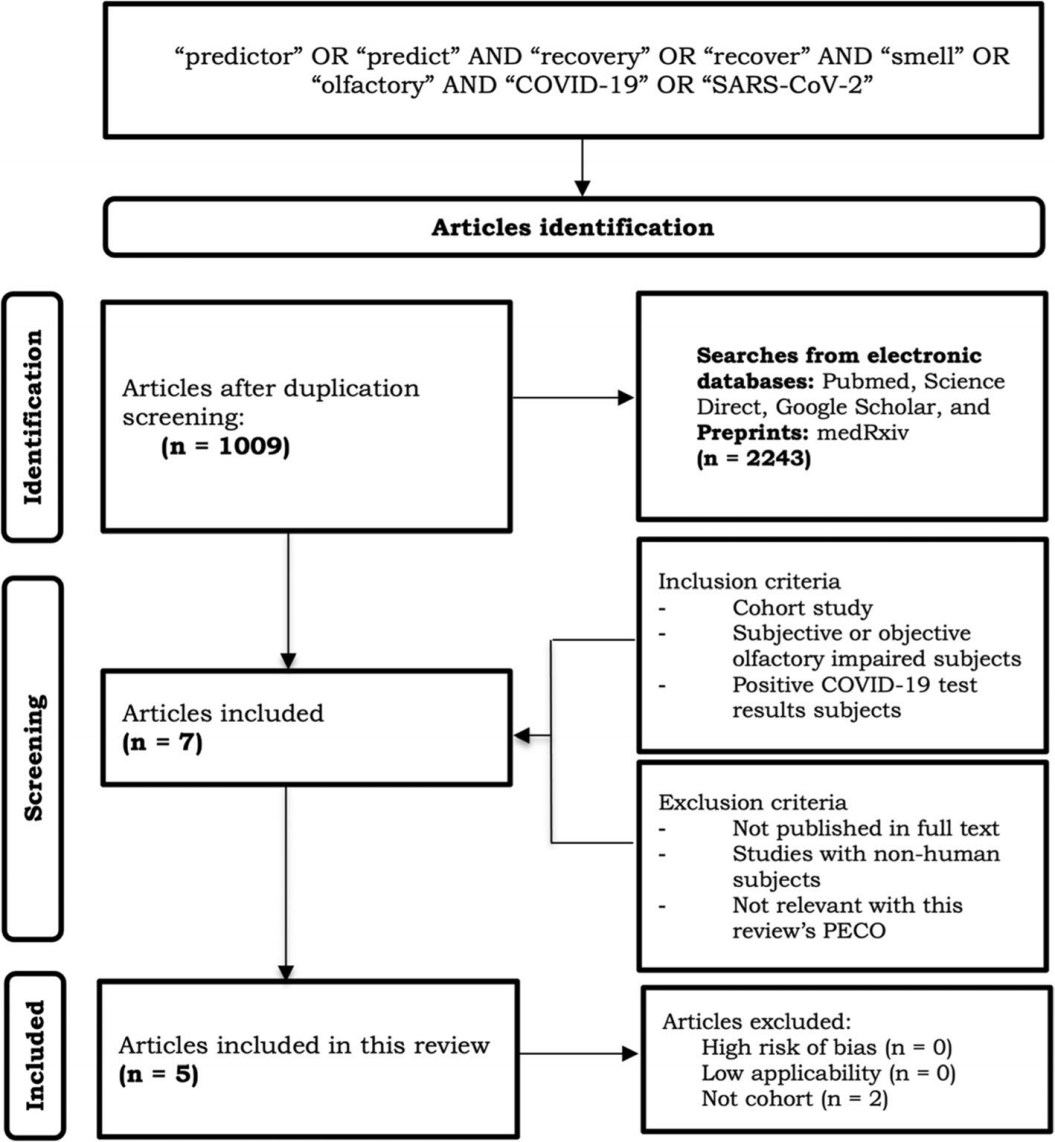
Flowchart of the literature review and retrieval based on PRISMA [13]

This review was carried out and reported based on the Preferred Reporting Items for Systematic Reviews and Meta-Analyses (PRISMA) 2020 [13] protocols and statements (Fig. 1). A preliminary literature search was undertaken only on March 15, 2022, and the completion date was on April 20, 2022. Relevant articles were identified in journal databases including PubMed, ScienceDirect, Google Scholar, and medRxiv for preprints. Searches were based on Patient/population, Exposure, Comparison/control, Outcome (PECO) as follows:

- P: people with COVID-19
- E: olfactory dysfunction
- C: people with COVID-19 without olfactory dysfunction
- O: olfactory function recovery

Searches were supplemented by hand searching. Reviewers also retrieve any additional literature in observational design meeting the eligibility criteria from literature references, preferably cohort studies to prevent possible poorly designed case-control studies.

### Eligibility criteria

The inclusion and exclusion criteria are as follows.

The following are the inclusion criteria:

- Cohort study
- The subjects of the study complained of olfactory dysfunction (all types of olfactory dysfunction) based on subjective or objective assessment (self-assessed or with any tools).
- The subjects are having or had COVID-19 during or before the occurrence of olfactory dysfunction.
- The studies were published from January 1, 2020, to April 15, 2022.
- The studies are published in English.

The following are the exclusion criteria:

- Not published in full text
- Studies with non-human subjects
- Not relevant with this review’s PECO

### Data collation

One reviewer will export the literature from the databases that match stated keywords to Zotero® to remove duplicates manually. The initial screening was done by scheming the appropriate title and abstract by two independent reviewers. Any literature that met the inclusion criteria and was exempted from the exclusion criteria will be rated and reviewed in full-text versions, and the articles will be retained. Data extracted from the literature are authors and year of the study, study title, study methods, statistical significance, and predictors (Table 1). Core components, including follow-up period, olfactory impairment recovery, and its measurements, are presented in Table 2.

**Table 1:** Data extraction from the literatures

Authors	Title	Method	Predictors	Significance (CI = 95%)
Amer et al. [14]	Early recovery patterns of olfactory disorders in COVID-19 patients; a clinical cohort study	*Cohort retrospective* in 96 subjects	Age 31–40 years Female	OR = 60.547; *p* ≤ 0.001 OR = 10.557; *p* = 0.005
Babaei et al. [15]	Factors associated with anosmia recovery rate in COVID-19 patients	*Cohort retrospective* in 235 subjects	Smoking Ageusia	OR = 10.813; *p* = 0.031 OR = 5.340; *p* ≤ 0.001
Coelho et al. [16]	Predictors of smell recovery in a nationwide prospective cohort of patients with COVID-19	*Cohort prospective* in 798 subjects	Age < 40 years Nasal congestion	*p* ≤ 0.003 *p* ≤ 0.03
Ferreli et al. [17]	Trigeminal features in COVID-19 patients with smell impairment	*Cohort retrospective* in 98 subjects	Trigeminal nasal sensations	OR = 6.176; *p* = 0.013
Teaima et al. [18]	Patterns and clinical outcomes of olfactory and gustatory disorders in six months: prospective study of 1031 COVID-19 patients	*Cohort prospective* in 1031 subjects	Parosmia	OR = 1.787; *p* = 0.0003

**Table 2:** Critical appraisal of the literature using PROBAST [12]

Study	Risk of bias				Applicability			Overall	
	**Participants**	**Predictors**	**Outcome**	**Analysis**	**Participants**	**Predictors**	**Outcome**	**Risk of bias**	**Applicability**
Amer et al. [14]	+	+	+	+	+	+	+	+	+
Babaei et al. [15]	+	+	+	+	+	+	–	+	+
Coelho et al. [16]	+	+	+	+	+	+	+	+	+
Ferreli et al. [17]	+	+	+	+	+	+	+	+	+
Teaima et al. [18]	+	+	+	+	+	+	+	+	+

*PROBAST* Prediction model Risk of Bias Assessment Tool

“ + ” indicates a low risk of bias/low concern regarding applicability; “–” indicates a high risk of bias/high concern regarding the applicability

### Quality assessment

Quality assurance was maintained by undergoing a critical appraisal of literature done by both reviewers. The PROBAST [12] checklist was used for all literature to assess the risk of bias, applicability, and overall relevancy (Table 3). Studies deemed as relevant are evaluated for this systematic review. Any disagreement will be resolved by consensus.

**Table 3:** Olfactory dysfunction prevalence, follow-up period, and assessment

Authors	Olfactory dysfunction results		Follow-up period	Olfactory dysfunction assessment
	**Recovered**	**Not recovered**		
Amer et al. [14]	72 (75%)	24 (25%)	1 month	DyNaCHRON [19] questionnaire
Babaei et al. [15]	207 (88.1%)	28 (11.9%)	2 months	Anosmia Reporting Tool
Coelho et al. [16]	634 (79.4%)	164 (20.6%)	6 months	Self-assessed
Ferreli et al. [17]	80 (81.6%)	18 (18.4%)	6 months	Sino-Nasal 22 Result Test (I-SNOT-22)
Teaima et al. [18]	909 (88.2%)	122 (11.8%)	6 months	Self-assessed

### Data synthesis

A narrative (descriptive) synthesis of the study findings will be provided. We will use aggregated patient data. We will thus examine the range over which prevalence varies in the literature. This will be reported in the extracted data table. We will provide an overview of the reported prevalence, olfactory testing methods, demographic data (gender, sex, age), comorbidities, and COVID-19-related symptoms the subject complained about. When in-group variances are correlated, we will consider the reported statistical methods and *p*-values. Based on these descriptions, we will provide our interpretation for further investigation needs for future studies.

## Results

### Search result

**Fig. 2:**
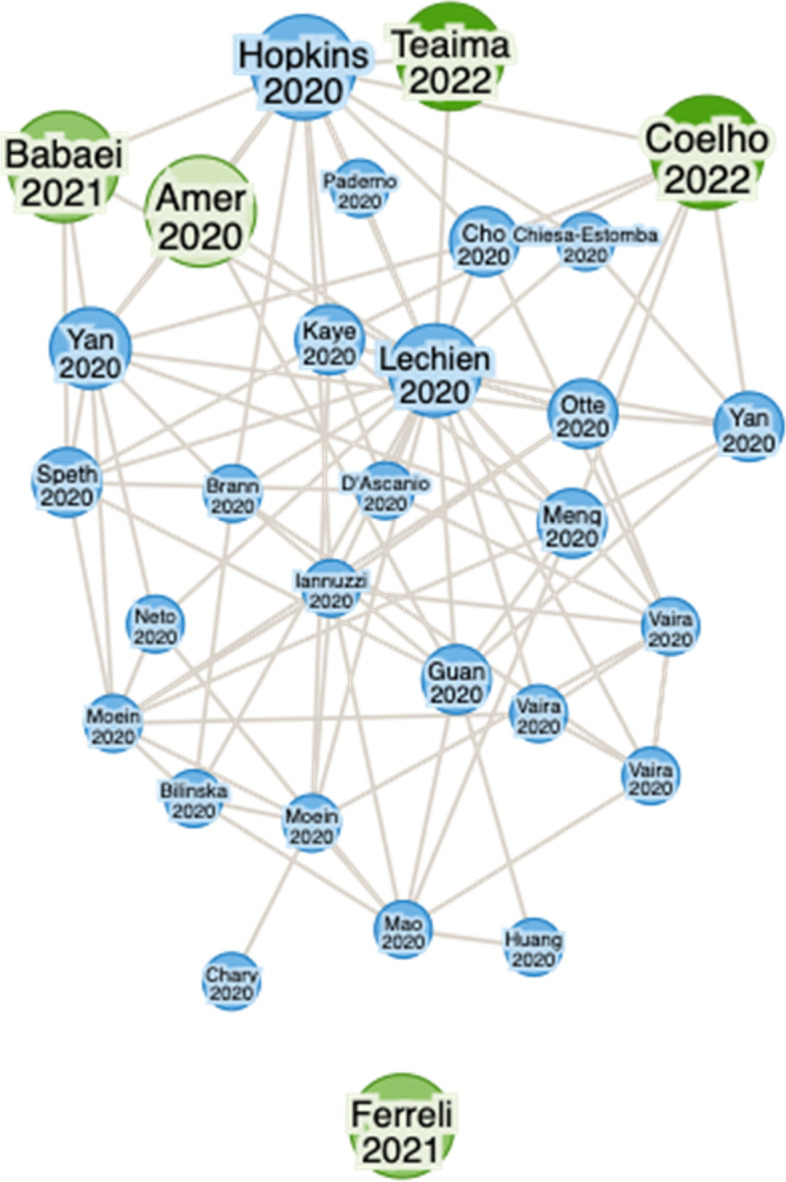
Research Rabbit literature mapping based on main journals

As many as 2243 citations were identified using the search strategy, of which a total of 1009 articles (300 from PubMed; 37 from Science Direct; 626 from Google Scholar; 46 from medRxiv) were screened after duplications were removed. After including the articles based on the inclusion criteria, only five articles were retrieved. Critical appraisal and risk of bias assessment were done on all five articles. We included publicly available studies and were linked to be similar work to the main five articles via the ResearchRabbit [20] literature mapping tool (Fig. 2) to support the main articles in narrating the review.

Of all 5 cohort studies, two were conducted in Egypt, and the rest were born in Iran, Italy, and the USA. This review yielded a total of 2259 subjects from a vast demographic background. Surprisingly, each study presented different predictive factors of olfaction recovery. The characteristics of each cohort are shown in Tables 1 and 3.

## Discussion

The impaired olfactory function can affect an individual’s quality of life, especially if the degree of impairment is moderate to severe. The prevalence of olfactory disorders worldwide is variable, with the lowest of 3% but can reach also reach as high as 22% [21–23].

Classically, olfactory disorders are divided into two categories, namely quantitative and qualitative olfactory impairment. Anosmia—complete olfactory dysfunction—is experienced by 3.6 to 5.8% of the world’s population, while hyposmia—partial olfactory dysfunction—has a greater prevalence (13–18%) [6]. Other terms, namely parosmia—a qualitative olfactory disorder, which is a distortion of recognizing an actual odor source—and phantosmia—distortion of smelling an odor without a source of stimuli—have a higher prevalence of 19% and 11%, respectively [7]. In addition, neurodegenerative disorders or toxins can cause olfactory dysfunction [6].

Another classification is acquired and congenital olfactory disorders, despite the latter being scarce in incidence. Three categories have been identified: (1) conductive dysfunction, (2) sensorineural dysfunction, and (3) central dysfunction [6]. Not all etiologies of acquired olfactory dysfunction can easily be classified into one of these three categories due to overlapping pathophysiologies in the mechanisms. The most common causes of acquired olfactory dysfunction were sinonasal disease (30%), upper respiratory tract infection (URTI, 25%), traumatic brain injury (TBI, 14%), or unknown (idiopathic, 12%) [24].

However, it is believed that the above rates do not reflect the true prevalence because the symptoms are not prominent compared to disturbances in other sensory functions [25] unless the olfactory disturbance is of a severe degree. One in 3 individuals with severe olfactory disorders will experience a significant decrease in their quality of life [6]. In addition to the predisposing factors above, an example of a current post-viral olfactory loss is olfactory impairment in COVID-19 patients, where respiratory and nasal olfactory cells express the angiotensin-converting enzyme 2 protein, which is used by the COVID-19 virus to infect cells or direct infection to the olfactory bulb [26]. Smell dysfunction in COVID-19 was reported in around 50% [27, 28] and up to 89.23% of individuals [29].

In this systematic review of predictive factors related to impaired olfaction in COVID-19 patients, we identified and critically appraised 5 studies. The predictive factors of each study are discussed as follows:

- Sex: Various studies have given varying results regarding the sex variable and its relationship with predictors of post-COVID-19 olfactory recovery. A higher incidence of anosmia in males [23] is proposed to be caused by the estradiol hormone which can play a protective role in the inflammatory process of olfactory function in female [9], but numerous studies also reported that olfactory dysfunction would likely persist in females [8, 10, 28, 29].
- Nasal congestion: Nasal obstruction was prevalent in around 20% [27, 30] and up to 67.8% of COVID-19 patients [4]. It is believed that nasal congestion causes “conductive” olfactory disorders due to coronavirus infection [16]. While a study stated nasal symptoms are statistically significant with smell and taste loss [28], this is in contrast to the study by Babaei et al. [15] which stated that the presence of nasal discharge was not correlated with olfactory dysfunction.
- Shortness of breath: Shortness of breath is often found in severe COVID-19 symptoms. Shortness of breath is one of the manifestations of disturbed air passage through the olfactory epithelium, so the lack of incoming stimuli causes olfactory function disorders [16]. If the symptoms of shortness of breath have been resolved, then the olfactory function will likely recover.
- Age: It is believed that those aged < 40 years have better resilience to tissue injury than older people [16]. Age > 70 years is a predictor of COVID-19 long-hauler [7], which could be related to recent studies and a meta-analysis that reported a higher prevalence of olfactory dysfunction in the older population [22, 29, 31]. Contrary to the previous study, age is postulated to not correlate with the recovery of olfactory function [10]. A cross-sectional study found that as the age increased by 1 year, the risk of olfactory and gustatory dysfunction increased by 5%; hypothesizing the incidence of said dysfunctions is higher in the younger generation [31].
- Ageusia: The sensation of smell and taste are so closely related that anosmia can cause dysgeusia or ageusia [23]. The previous study denotes the finding in 83% of patients with COVID-19 symptoms [29]. The recommended pathophysiology for dysgeusia in COVID-19 is cranial nerve dysfunction (especially the seventh cranial nerve), zinc deficiency, interaction with sialic acid receptors, and direct viral attack on buccal and gingival tissues, taste buds on the tongue, and salivary glands [32].
- Parosmia: Parosmia—distortion in one’s ability to smell with known odorant—is experienced in many COVID-19 survivors. One study presented the nature of parosmia as the most prevalent in as early as 3 months until 1 year after COVID-19 symptoms onset [7]. Studies denoting its correlation with recovery showed various results, including a study by Makaronidis et al. [9], that reported parosmia worsens olfactory function after COVID-19. It is believed that parosmia is caused by decreased repair of olfactory neurons in the neural circuit, so there is a possibility of the emergence of immature neurons[33]; in previous studies, parosmia showed a poor prognosis [9]. Unfortunately, in a recent study, nearly half of COVID-19 survivors complained of parosmia after one and a half years [5]. Therefore, parosmia in the context of post-viral olfactory disorders cannot be concluded.
- Trigeminal sensation: Significant results were obtained from the olfactory function test using the Sniffin’ Sticks Test in the study by Ferreli et al. [17]. These findings suggest that intranasal trigeminal nerve endings may also be potential targets of SARS-CoV-2 infection and the olfactory neuroepithelium. The same is true of other studies of respiratory neurotrophic viruses [34]. Several researchers have hypothesized that the central mechanism of trigeminal damage occurs due to the connection of the olfactory system with the trigeminal nerve itself, with overlapping activation in areas such as the piriform cortex, ventral insula, and medial frontal gyrus [35].
- Cigarette smoking: Cigarette smoking is a predictor of recovery of olfactory function 4 weeks after coronavirus infection [15]. Several researchers have cited lower rates of COVID-19 infection among individual smokers. They observed this with two hypotheses. The first hypothesis considers a global effect on the renin-angiotensin system, of which ACE2 is only a part, by regulating ACE1 receptors and downregulating ACE2 receptors. The second theory is the interaction between ACE2 and nicotinic (primarily nicotine seven receptors). This receptor is near the ACE2 receptor on the cell membrane. Dysregulation of these receptors may trigger a Th1 immune response. Nicotine and SARS-Cov2 may also compete for binding to nicotinic acetylcholine receptors (nAchRs) [36]. However, research on this variable is still controversial. Several cross-sectional studies yielded no significant result between the prevalence of anosmia and smoking habit [23, 30], but another study stated the contrary [28]. Previous cross-sectional studies also proposed several predictive factors relating to the olfactory sequelae:
- Dental flossing: Daily flossing was shown to significantly reduce clinical gingival inflammation and bleeding [37], which suggests flossing with better gingival health and decreases chances of inflammation. Mechanically, this study is related to previous hypotheses regarding local mechanisms of COVID-19 infection, such as viral infiltration of locally inflamed periodontal pockets [38]. But we recommend a cautious interpretation of the association between flossing and sensory recovery, as it cannot be interpreted as causative. For example, flossing may be a weak indicator of a more compliant and health-focused individual as the clinical significance cannot be automatically assumed, although the difference in flossers between smell-recovery groups (75% of rapid smell recovery vs. 58% of prolonged smell recovery) was statistically significant.
- Platelet count: In a study by Ardestani et al. [39], platelet count was the most important predictor in the recovery process of olfactory function in post-COVID-19 patients. As mentioned above, some coronavirus particles can infect endothelial cells due to the role of the ACE2 receptor. This endothelium—especially in the small blood vessels—with slower blood flow can cause thrombosis in the microcirculatory system. This mechanism may lead to thrombus formation, as reported by Xin Zhou et al. [40]. Therefore, patients who have lower platelets have a lower risk of thrombosis in the vessels of the olfactory system. Antiplatelet drugs as prophylactic agents for olfactory disorders due to COVID-19 may help inhibit platelet activation in individuals with high platelet counts [39]. However, due to the study method, missing data and errors throughout data collection and follow-up in this multicentric cross-sectional study by different practitioners from other cities were unavoidable.

### Study strengths and limitations

The evidence on exact predictive factors on olfactory healing is less striking, mainly due to the limited number of studies and problems related to the methodological quality of the studies. Thus, the predictive factors mentioned above appear controversial and need to be further assessed as appraised prediction models. Unfortunately, we did not find any data on the exact prediction models of different outcomes, especially the smell recovery in post-COVID-19 patients, regardless of the COVID-19 infection recovery durations, different follow-up periods, or different demographic.

### Future application and research

This systematic review presented results that can be further applied to formulate strategies to address problems related to olfactory function post-COVID-19 infection, thus improving the effectiveness of treatment outcomes. Based on the above predictive factors, we suggest the implementation of the following approach:

1. Assess the patient’s background, including activities of daily living that can be related to one’s olfactory outcome.
2. Physicians should develop an exact quantitative or reporting measure that could be proposed nationwide to help patients suffering from olfactory alteration to understand better their disability seen from patients’ probability of underreporting olfactory dysfunction.
3. Educate patients about the outcome and the prognosis of the olfactory dysfunction and what could be done to improve the olfactory function
4. Offer therapeutic interventions to vulnerable groups of people who are likely to benefit from it.

## Conclusion

Based on this systematic review, there is limited evidence to predict the recovery of olfactory function in people infected by coronavirus (COVID-19). The factors that consistently predict the recovery of olfactory function have not been established. Still, most studies have shown that age, sex, and several specific COVID-19 symptomatologies affect olfactory outcomes after COVID-19. Future studies reporting the predictor of olfactory dysfunction in subjects with or post-COVID-19 and the possible confounding factors should incorporate multiple control populations to help us answer this question which could be implemented in further management in dealing with post-COVID-19 olfactory sequelae.

## Data Availability

The datasets used in this study are available from the corresponding author upon reasonable request.
